# Low participation in voluntary period for patient-reported outcome performance measures for hip and knee replacement

**DOI:** 10.1093/haschl/qxaf111

**Published:** 2025-05-30

**Authors:** Nicholas L Berlin, Zoey Chopra, Caroline Thirukumaran, Thomas C Tsai, David W Bates, Andrea L Pusic, Jason B Liu

**Affiliations:** Department of Surgery, Brigham and Women's Hospital, Boston, MA 02115, United States; University of Michigan School of Medicine, Ann Arbor, MI 48109, United States; Department of Orthopedic Surgery, Northwestern University School of Medicine, Chicago, IL 60611, United States; Department of Surgery, Brigham and Women's Hospital, Boston, MA 02115, United States; Division of General Internal Medicine and Primary Care, Brigham and Women's Hospital, Boston, MA 02115, United States; Division of General Internal Medicine and Primary Care, Brigham and Women's Hospital, Boston, MA 02115, United States; Department of Surgery, Brigham and Women's Hospital, Boston, MA 02115, United States; Patient-Reported Outcomes, Value, and Experience (PROVE) Center, Brigham and Women's Hospital, Boston 02120, MA, United States; Department of Surgery, Brigham and Women's Hospital, Boston, MA 02115, United States; Patient-Reported Outcomes, Value, and Experience (PROVE) Center, Brigham and Women's Hospital, Boston 02120, MA, United States

**Keywords:** patient-reported outcomes, quality measurement, Medicare, knee replacement, hip replacement

## Introduction

Since July 1, 2024, hospitals have been mandated by the Centers for Medicare and Medicaid Services (CMS) to collect patient-reported outcome-based performance measures (PRO-PMs) for Medicare fee-for-service (FFS) beneficiaries undergoing inpatient total hip and knee arthroplasties (THA and TKA, respectively).^1^ Hospitals that fail to submit a complete dataset for at least 50% of all eligible patients, which includes prespecified patient-reported outcome measures (PROMs) (including the HOOS Jr. and KOOS Jr. scales) within 3 months prior to surgery and between 300 and 425 days postoperatively. Hospitals that do not comply will be penalized with reduced payments for all Medicare FFS Part A claims, including nonorthopedic claims, and disqualified from value-based purchasing programs. For example, a hospital with $100 million annually of Medicare Part A claims failing to comply faces an annual penalty of approximately $1 million.^2^ Even well-resourced hospitals have reported difficulty achieving the minimum 50% response rate.^3^ Acknowledging this resource-intensive paradigm shift for more than 700 000 patients who undergo these surgeries annually,^4^ CMS provided hospitals subject to this regulation with a voluntary preparatory period to submit data and receive confidential feedback to improve data-collection processes, support patients, and improve outcomes. In this study, we investigated hospital-level rates of voluntary reporting of PRO-PMs during the preparatory period, which may provide insight into hospital readiness to comply with these mandates.

## Methods

We performed a cross-sectional analysis of publicly available data from CMS regarding THA and TKA PRO-PM collection during the preparatory period from January 1 to June 30, 2023.^5^ Hospitals that voluntarily collected PROMs during the Comprehensive Care for Joint Replacement model were also considered to have experience collecting PRO-PMs.^6^ Hospitals participating in the forthcoming mandatory bundled payment model, Transforming Episode Accountability Model (TEAM), which also requires PRO-PM reporting, were identified. The sample was limited to Medicare Inpatient Prospective Payment System (IPPS) hospitals, and characteristics were obtained from the American Hospital Association Annual Survey. Unadjusted bivariate analyses were performed to compare characteristics of hospitals that did and did not participate. Statistical significance was set at α = .05 and all tests were 2-sided.

## Results

Of the 2920 hospitals included, 101 hospitals (4%) voluntarily reported PRO-PMs (Table 1). Among hospitals selected to participate in the forthcoming TEAM model, 28 hospitals (4%) voluntarily reported PRO-PMs. In unadjusted analyses of IPPS hospitals, hospitals that voluntarily reported were more often larger (24% vs 16% with 400+ beds; *P* = .015), not-for-profit (85% vs 66%; *P* < .001), teaching (83% vs 63%; *P* < .0.001), and safety-net (34.7% vs 25.9%; *P* = .048) hospitals located in an urban setting (90% vs 77%; *P* = .002) in the Northeast region (37% Northeast vs 28% Midwest vs 26% South vs 10% West; *P* < .001) (Table 1). Hospitals that voluntarily reported were more likely to have higher health care utilization, including higher mean annual hospital admissions (15 228 vs 9794; *P* < .001), outpatient visits (381 388 vs 231 287; *P* < .001), and surgical operations (14 744 vs 8135; *P* < .001) (Table 1). Mean annual Medicare inpatient discharges were higher for hospitals that reported (7082 vs 4415; *P* < .001).

**Table 1.** US hospital characteristics associated with voluntary reporting of total hip and knee replacement patient-reported outcome-based performance measures.

	All IPPS hospitals			
	Collection of PRO-PMs			
	No	Yes	Total	*P*
Hospitals, No. (%)	2819 (96.5%)	101 (3.5%)	2920	
TEAM participation, No.(%)				
No	2141 (75.9%)	73 (72.3%)	2214 (75.8%)	.397
Yes	678 (24.1%)	28 (27.7%)	706 (24.2%)	
Region, No. (%)				
Northeast	404 (14.3%)	37 (36.6%)	441 (15.1%)	<.001
Midwest	652 (23.1%)	28 (27.7%)	680 (23.3%)	
South	1191 (42.2%)	26 (25.7%)	1217 (41.7%)	
West	572 (20.3%)	10 (9.9%)	582 (19.9%)	
Teaching status, No. (%)				
Non-teaching	1051 (37.3%)	17 (16.8%)	1068 (36.6%)	<.001
Teaching	1768 (62.7%)	84 (83.2%)	1852 (63.4%)	
Safety-net hospital, No. (%)				.048
No	2090 (74.1%)	66 (65.3%)	2156 (73.8%)	
Yes	729 (25.9%)	35 (34.7%)	764 (26.2%)	
Urbanicity, No. (%)				
Rural	658 (23.3%)	10 (9.9%)	668 (22.9%)	.002
Urban	2161 (76.7%)	91 (90.1%)	2252 (77.1%)	
Ownership type, No. (%)				
Government	367 (13.0%)	5 (5.0%)	372 (12.7%)	<.001
Nonprofit	1858 (65.9%)	86 (85.1%)	1944 (66.6%)	
For-profit	594 (21.1%)	10 (9.9%)	604 (20.7%)	
Number of beds, No. (%)				
<200 beds	1671 (59.3%)	46 (45.5%)	1717 (58.8%)	.015
200-399 beds	706 (25.0%)	31 (30.7%)	737 (25.2%)	
400+ beds	442 (15.7%)	24 (23.8%)	466 (16.0%)	
Hospital admissions, mean (SD)	9794 (10 587)	15 228 (15 391)	9987 (10 831)	<.001
Inpatient days, mean (SD)	55 705 (67 037)	86 938 (95 691)	56 785 (68 445)	<.001
Outpatient visits, mean (SD)	231 287 (346 789)	381 388 (45 172)	236 479 (351 915)	<.001
Surgical operations, mean (SD)	8135 (9599)	14 744 (19 531)	8364 (10 173)	<.001
Inpatient discharges, mean (SD)	9884 (10 728)	15 530 (15 562)	10 079 (10 975)	<.001
Medicare inpatient discharges, mean (SD)	4415 (4530)	7082 (6449)	4508 (4634)	<.001
Medicaid inpatient discharges, mean (SD)	2168 (2859)	3270 (3886)	2206 (2907)	<.001
Medicare inpatient discharge share, mean (SD)	0.5 (0.1)	0.5 (0.1)	0.5 (0.1)	.192
Medicaid inpatient discharge share, mean (SD)	0.2 (0.1)	0.2 (0.1)	0.2 (0.1)	.294
Disproportionate patient share, mean (SD)	0.7 (0.1)	0.7 (0.1)	0.7 (0.1)	.121

Source: Authors’ analysis of publicly available data from CMS regarding voluntary THA and TKA PRO-PMs among IPPS hospitals collection as of October 2024. Hospital characteristics were obtained from the 2022 American Hospital Association Annual Survey. *P* values represent statistical comparisons between hospitals with and without collection of PRO-PMs with chi-square tests for categorical variables or *t* tests for continuous variables.

Abbreviations: CMS, Centers for Medicare and Medicaid Services; IPPS, Inpatient Prospective Payment System; PRO-PM, patient-reported outcome performance measure; TEAM, Transforming Episode Accountability Model; THA, total hip arthroplasty; THK, total knee arthroplasty.

## Discussion

PRO-PMs are a critical component of future CMS payment reforms, enhancing the incorporation of the patient's perspective into quality measurement.^7^ Our data highlight low rates of hospital participation in the voluntary preparatory period ahead of CMS mandates. The reasons for low participation may be related to several factors, including a lack of readiness among hospitals, low perceived value of voluntary reporting, or preferred allocation of limited resources towards strategic preparation for the upcoming mandates. If low participation rates are due to a lack of readiness, then our data underscore widespread changes that hospitals must implement to comply with these mandates. Even if 50% of hospitals were fully prepared and elected not to participate, then nearly half of the hospitals were either unprepared or rapidly scaling these changes.

Although we assume that hospitals that did not voluntarily participate were not prepared to report, collecting PROMs with high fidelity is challenging due to systems-level factors such as gaps in infrastructure, implementation costs, and workflow barriers, as well as patient-level factors such as lack of knowledge and low perceived value of completing surveys.^8-11^ Our data demonstrate how hospitals with fewer resources were less likely to participate and therefore were less likely to receive feedback to improve their data-collection processes. This may place these hospitals at higher risk of financial penalties for noncompliance. Given the financial investment and clinical transformation needed to gather PRO-PMs,^8-11^ The CMS may consider interventions to protect these hospitals, including waivers for specific categories of hospitals to avoid financial penalties, sharing best practices for data collection, and innovative partnerships with professional organizations such as the American Academy of Orthopedic Surgeons to improve uptake.^12^ Future policies that mandate collection of PROMs for PRO-PMs should strengthen support for low-resourced hospitals prior to mandated collection with financial risk.

Limitations include the cross-sectional nature of this study and small sample size, which preclude causal relationships and precluded our ability to perform adjusted analyses.

## Supplementary Material

qxaf111_Supplementary_Data

## Data Availability

Data is publicly available through the Centers for Medicare and Medicaid Services.
